# Time interval between breast cancer diagnosis and surgery is associated with disease outcome

**DOI:** 10.1038/s41598-023-39259-3

**Published:** 2023-07-26

**Authors:** Siji Zhu, Shuai Li, Jiahui Huang, Xiaochun Fei, Kunwei Shen, Xiaosong Chen

**Affiliations:** 1 Department of General Surgery, Comprehensive Breast Health Center, Ruijin Hospital, Shanghai Jiaotong University School of Medicine, Shanghai, 200025 China; 2 Department of Pathology, Ruijin Hospital, Shanghai Jiaotong University School of Medicine, Shanghai, 200025 China

**Keywords:** Breast cancer, Oncology

## Abstract

Time interval between breast cancer (BC) diagnosis and surgery is of concern to patients and clinicians, but its impact on survival remains unclear. We identified 5130 BC patients receiving surgery between 2009 and 2017 from the Shanghai Jiaotong University Breast Cancer Database (SJTU-BCDB), and divided as Ruijin cohort and SJTU cohort. All participants were divided into three groups according to the interval between diagnosis and surgery: ≤ 1 week, 1–2 weeks, and > 2 weeks. Among 3144 patients of Ruijin cohort, the estimated 5-year breast cancer-free interval (BCFI) rates for the ≤ 1 week, 1–2 weeks and > 2 weeks groups were 91.8%, 87.5%, and 84.0% (P = 0.088), and the estimated 5-year overall survival (OS) rates were 95.6%, 89.6%, and 91.5% (P = 0.002). Multivariate analysis showed that patients with a TTS > 2 weeks had significantly lower BCFI (HR = 1.80, 95%CI 1.05–3.11, P = 0.034) and OS (HR = 2.07, 95% CI 1.04–4.13, P = 0.038) rates than patients with a TTS ≤ 1 week. Among 5130 patients when combining Ruijin cohort with SJTU cohort, the estimated 5-year BCFI rates for the ≤ 1 week, 1–2 weeks, and > 2 weeks groups were 91.0%, 87.9%, and 78.9%, and the estimated 5-year OS rates for the ≤ 1 week, 1–2 weeks, and > 2 weeks groups were 95.8%, 90.6%, and 91.5%, both with a significantly p value < 0.001. Our findings demonstrated the prolonged time to surgery (more than 2 weeks) after BC diagnosis was associated with poor disease outcomes, suggesting that efforts to early initiate treatment after diagnosis need to be pursued where possible to improve survival.

## Introduction

Timely surgery after disease diagnosis is theoretically ideal in cancer treatment, and some guidelines recommend a maximum wait time of 2–4 weeks for all cancer operations^1^. For breast cancer (BC), since early detection can reduce mortality^2^, it is reasonable that efforts to shorten the interval from diagnosis to definite surgery may improve the prognosis of BC patients.

However, a variety of patient-, provider-, and health system-related factors may contribute to the delay of treatment initiation^3–6^, and the optimal time to surgery (TTS) for BC is still unclear. Several studies were conducted to assess whether a prolonged TTS is associated with a poorer prognosis, but the results were conflicting^7–9^. Furthermore, these intervals described in previous studies were all calculated on a monthly basis, which were different from the current situation of Chinese cancer care: Due to the large patient population, the Chinese cancer care system should accelerate treatment procedure to shorten the hospitalization period time, and the surgery initiation after breast cancer diagnosis is relatively faster than western countries^10,11^.

Besides the probable impact on survival, long interval from diagnosis to definite surgery will also bring great anxiety to patients. Therefore, knowing the potential influence of long TTS on patients’ survival and distinguishing the subgroup of patients who need more prompt treatment are clinically valuable, especially in unexpected situations such as the outbreak of coronavirus disease 2019 (COVID-19), which poses a major challenge to the health care system in most areas of the world and leads to the suspension of selected invasive procedures to protect patients and health care workers, thus to conserve hospital resources^12–14^.

Hence, we aimed to evaluate the prolonged time to surgery in two large consecutive cohorts receiving adjuvant treatment of modern era, to identify potential sociodemographic and clinicopathological factors for prolonged TTS, and then to establish the association between prolonged TTS and prognosis.

## Methods

### Study population

Patients who received a pathologic diagnosis of primary BC were identified from the Shanghai Jiaotong University Breast Cancer Database (SJTU-BCDB), which is a prospectively maintained web-based database containing 40 breast cancers centers. Patients treated in Comprehensive Breast Health Center, Ruijin Hospital were identified as the Ruijin cohort. Patients treated in other breast cancer centers except Ruijin hospital, were identified as the SJTU cohort. Eligible patients were women diagnosed with stage I–III tumors between January 2009 and December 2017. Patients met with following criteria were excluded: undergoing surgery for in situ carcinoma, undergoing surgery directly without pathological biopsy, receiving neoadjuvant treatment, and bilateral BC.

All procedures performed in studies involving human participants were approved by the independent Ethical Committees of Ruijin hospital. All clinical information was obtained and approved by SJTU-BCDB database.

### Patient’s clinicopathological features

The collected data included patients’ sociodemographic factors (residence area, education level), clinicopathological characteristics (age, menopausal status, comorbid condition, tumor stage, pathological type, histologic grade, body mass index (BMI), hormone receptor (HR) status, HER2 status, lymphovascular invasion (LVI), Ki67 index, and molecular subtype) and details of treatment (breast surgery, radiotherapy, chemotherapy, endocrine therapy, and HER2-targeted therapy). Tumor stage was based on pathologic criteria according to the seventh edition of the American Joint Committee on Caner (AJCC) TNM staging system^15^. Comorbid condition was evaluated by using the Charlson Comorbidity index (CCI) and divided into 0, 1–2 or 3 +^16^. Prolonged time to chemotherapy (TTC) was defined as the interval from surgery to chemotherapy > 6 weeks. Prolonged time to radiotherapy (TTR) was defined as interval from surgery to radiotherapy > 32 weeks for those patients receiving chemotherapy, or > 12 weeks for those patients not receiving chemotherapy.

### Interval between diagnosis and surgical treatment initiation

The interval between diagnosis and surgical treatment initiation was defined as the time from pathological diagnosis to the definite surgery. Pathological diagnosis was made by core needle biopsy (CNB). The interval of TTS was categorized as ≤ 1 week, 1–2 weeks, and > 2 weeks.

The diagnostic and surgical procedures are as followed: Patients with suspicious breast lesion will be scheduled for hospitalization after outpatient consultation. After the hospitalization, the radiologists will involve to perform image assessments and lesion localization. Patients will receive core needle biopsy after image assessments finished. Breast surgeons do the core needle biopsy. The primary pathological results will be presented in 1–2 days after CNB by our pathologists with malignant or benign diagnosis. The receptor status by Immunohistochemistry (IHC) testing will be reported in another 2 days after primary diagnosis result. Majority of patients will receive surgery after receiving pathological diagnosis with or without IHC result.

### Follow-up

For all patients, outpatient visits or telephone calls were performed every 3 to 6 months until death. The primary endpoint was the breast cancer-free interval (BCFI), which was defined as the length of time from surgery to the first occurrence of the following events: locoregional recurrence of any invasive disease, contralateral invasive BC, distant recurrence, and BC-related death. The secondary endpoint was overall survival (OS), which was defined as the length of time from surgery to any cause of death.

### Statistical analyses

Distributions of patient sociodemographic, clinicopathological, and treatment characteristics by TTS intervals were examined using χ^2^ or Fisher’s exact tests. We evaluated the association between TTS and survival using Cox proportional hazards regression models. Prognostic factors with significant or marginal p values (P < 0.1) in the univariate analysis were included in the multivariate analysis. Planned subgroup analyses included the Cox models according to the age at diagnosis, molecular subtype, tumor stage and radiotherapy. Two-sided P values < 0.05 were considered statistically significant. Statistical analyses were conducted with IBM SPSS version 20 (SPSS Inc., Chicago, IL, USA). We performed propensity score matched (PSM) analysis in the combination of Ruijin & SJTU cohort by using R program version 3.6.3. The command matched 5 patients with TTS ≤ 1 week to one patient with 1–2 weeks and one patients with TTS > 2 weeks using factors including age, CCI, tumor stage, molecular subtype, pathological type, tumor grade and surgery type, and the caliper value of PSM was 0.2.

### Ethical approval

All data was obtained from SJTU-BCDB database. This study was conducted in accordance with the Declaration of Helsinki, and approved by the independent Ethical Committees of Ruijin hospital. Given the anonymised nature of the data, the requirement for informed consent was waived by the independent Ethical Committees of Ruijin hospital.

## Results

### The Ruijin cohort

#### Patient characteristics

**Figure 1:**
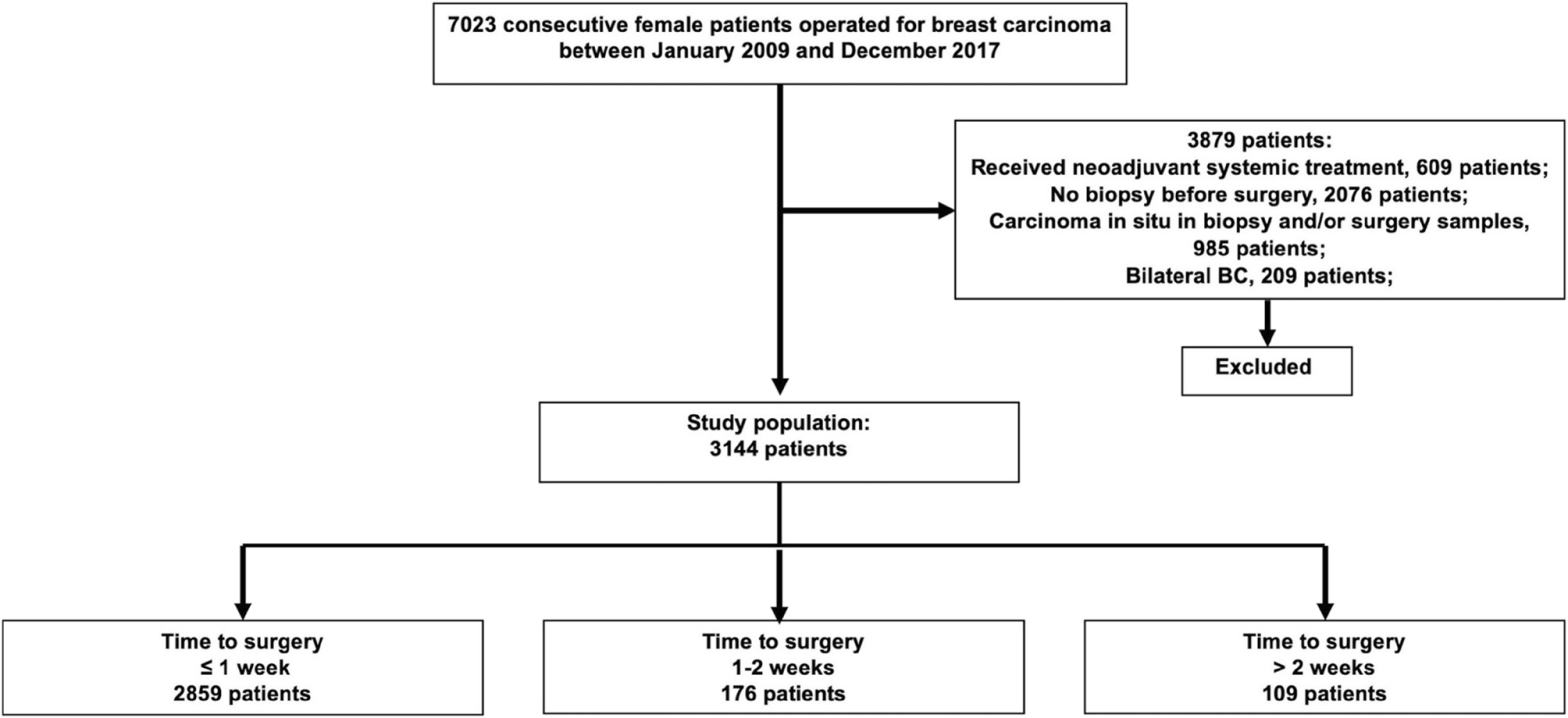
Identification of study population, the Ruijin cohort.

A total of 7023 patients underwent curative surgery for BC at Ruijin Hospital between January 2009 and December 2017. Finally, 3144 patients were included (Fig. 1). The median time from pathological diagnosis to surgery was 4 days (range from 0 to 59 days) (Figs. S1, S2a). Patients who received surgery within ≤ 1 week, 1–2 weeks, and > 2 weeks after diagnosis accounted for 90.9%, 5.6%, and 3.5% of all patients, respectively.

#### Association between TTS and clinicopathological characteristics

Descriptive results according to TTS are detailed in Table 1. The mean age of all participants in this study was 55.9 years. Regarding the demographic characteristics, patients with a prolonged interval (> 2 weeks) were more likely to be aged > 70 years old (P < 0.001) and to have CCI more than 3 (P < 0.001). Residence area, education level, and BMI were not associated with a prolonged interval between diagnosis and treatment initiation (P > 0.05). When calculating the change in the Ki67 index by TTS, patients with TTS greater than 2 weeks had a significantly higher Ki67 increase than patients with TTS less than 2 weeks (7.3% vs. 4.0%, P = 0.022) (Fig. S3). In addition, clinicopathological features, such as tumor stage, tumor grade, operation type, and molecular subtype, also did not differ according to TTS.

**Table 1:** Baseline clinical and pathological characteristics by time to surgery after diagnosis, The Ruijin cohort.

Characteristics	Total N = 3144 (%)	≤ 1week N = 2859 (%)	1–2 weeks N = 176 (%)	> 2 weeks N = 109 (%)	*P* value
Age (y/o)					
< 40	277 (8.8)	236 (8.3)	28 (15.9)	13 (11.9)	< 0.001
40–49	719 (22.9)	642 (22.5)	53 (30.1)	24 (22.0)	
50–70	1765 (56.1)	1647 (57.6)	69 (39.2)	49 (45.0)	
> 70	383 (12.2)	334 (11.7)	26 (14.8)	23 (21.1)	
Menstrual status					
Pre/Peri-	1132 (36.0)	1002 (35.0)	91 (51.7)	39 (35.8)	< 0.001
Post-	1998 (63.6)	1847 (64.7)	84 (47.7)	67 (61.5)	
NA	14(0.4)	10(0.3)	1(0.6)	3(2.8)	
Residence area					
Large metropolitan	1813 (57.7)	1650 (57.7)	96 (54.5)	67 (61.5)	0.845
Metropolitan	114 (3.6)	104 (3.6)	6 (3.4)	4 (3.7)	
Urban	337 (10.7)	313 (10.9)	17 (9.7)	7 (6.4)	
Rural	787 (25.0)	708 (24.8)	51 (29.0)	28 (25.7)	
NA	93 (3.0)	84 (2.9)	6 (3.4)	3 (2.8)	
CCI					
0	1855 (59.0)	1701 (59.5)	111 (63.1)	43 (39.4)	< 0.001
1–2	967 (30.8)	878 (30.7)	46 (26.1)	43 (39.4)	
3 +	322 (10.2)	280 (9.8)	19 (10.8)	23 (21.1)	
Education level					
Post-graduated	49 (1.6)	46 (1.6)	3 (1.7)	0 (0)	0.227
Degree	788 (25.1)	702 (24.6)	52 (29.5)	34 (31.2)	
High school	964 (30.7)	887 (31.0)	48 (27.3)	29 (26.6)	
Middle school	1161 (36.9)	1065 (37.3)	60 (34.1)	36 (33.0)	
NA	182 (5.8)	159 (5.6)	13 (7.4)	10 (9.2)	
BMI					
< 25	2228 (70.9)	2027 (70.9)	131 (74.4)	70 (64.2)	0.287
25–30	737 (23.4)	673 (23.5)	32 (18.2)	32 (29.4)	
≥ 30	114 (3.6)	103 (3.6)	8 (4.5)	3 (2.8)	
NA	65 (2.1)	56 (2.0)	5 (2.8)	4 (3.7)	
Breast surgery					
BCS	864 (27.5)	793 (27.7)	46 (26.1)	25 (22.9)	0.501
Mastectomy	2280 (72.5)	2066 (72.3)	130 (73.9)	84 (77.1)	
Axillary surgery					
SLNB	1727 (54.9)	1572 (55.0)	92 (52.3)	63 (57.8)	0.617
ALND	1405 (44.7)	1279 (44.7)	82 (46.6)	44 (40.4)	
NA	12(0.4)	8(0.3)	2(1.1)	2(1.8)	
Histology type					
IDC	2760 (87.8)	2511 (87.8)	156 (88.6)	93 (85.3)	0.690
Non-IDC	384 (12.2)	348 (12.2)	20 (11.4)	16 (14.7)	
Tumor size					
≤ 2.0 cm	1623 (51.6)	1490 (52.1)	81 (46.0)	52 (47.7)	0.227
> 2.0 cm	1519 (48.3)	1368 (47.8)	94 (53.4)	57 (52.3)	
NA	2(0.1)	1(0.1)	1(0.6)	0	
ALN status					
Negative	1947 (61.9)	1773 (62.0)	107 (60.8)	67 (61.5)	0.944
Positive	1197 (38.1)	1086 (38.0)	69 (39.2)	42 (38.5)	
Tumor stage					
I	1134 (36.1)	1041 (36.4)	59 (33.5)	34 (31.2)	0.238
II	1539 (49.0)	1384 (48.4)	91 (51.7)	64 (58.7)	
III	471 (15.0)	434 (15.2)	26 (14.8)	11 (10.1)	
Histological grade					
I	164 (5.2)	142 (5.0)	10 (5.7)	12 (11.0)	0.153
II	1549 (49.3)	1413 (49.4)	89 (50.6)	47 (43.1)	
III	1083 (34.4)	990 (34.6)	58 (33.0)	35 (32.1)	
NA	348 (11.1)	314 (11.0)	19 (10.8)	15 (13.8)	
LVI					
Negative	2831 (90.0)	2577 (90.1)	154 (87.5)	100 (91.7)	0.438
Positive	313 (10.0)	282 (9.9)	22 (12.5)	9(8.3)	
ER					
Negative	887 (28.2)	800 (28.0)	55 (31.2)	32 (29.4)	0.623
Positive	2257 (71.8)	2059 (72.0)	121 (68.8)	77 (70.6)	
PR					
Negative	1302 (41.4)	1175 (41.1)	81 (46.0)	46 (42.2)	0.430
Positive	1842 (58.6)	1684 (58.9)	95 (54.0)	63 (57.8)	
HER2					
Negative	2332 (74.2)	2112 (73.9)	130 (73.9)	90 (82.6)	0.125
Positive	812 (25.8)	747 (26.1)	46 (26.1)	19 (17.4)	
Ki67					
< 14%	953 (30.3)	869 (30.4)	54 (30.7)	30 (27.5)	0.810
≥ 14%	2191 (69.7)	1990 (69.6)	122 (69.3)	79 (72.5)	
Molecular subtype					
HR + /HER2 −	1877 (59.7)	1703 (59.6)	105 (59.7)	69 (63.3)	0.275
HER2 +	812 (25.8)	747 (26.1)	46 (26.1)	19 (17.4)	
TNBC	455 (14.5)	409 (14.3)	25 (14.2)	21 (19.3)	

The relationships between TTS and adjuvant treatment are shown in Table 2. Statistical significance was only found between prolonged interval and adjuvant chemotherapy (P = 0.009). And there were not statistical significant relationships between TTS and prolonged TTC (P = 0.120) nor prolonged TTR (P = 0.567).

**Table 2:** Adjuvant systemic therapy by time to surgery after diagnosis, The Ruijin cohort.

Characteristics	Total N = 3144 (%)	≤ 1week N = 2859 (%)	1–2 weeks N = 176 (%)	> 2 weeks N = 109 (%)	*P* value
Chemotherapy					
No	834 (26.5)	740 (25.9)	52 (29.5)	42 (38.5)	0.009
Yes	2307 (73.4)	2116 (74.0)	124 (70.5)	67 (61.5)	
NA	3 (0.1)	3 (0.1)	0 (0)	0 (0)	
Radiation therapy					
No	1509 (48.0)	1359 (47.5)	91 (51.7)	59 (54.1)	0.246
Yes	1632 (51.9)	1497 (52.4)	85 (48.3)	50 (45.9)	
NA	3 (0.1)	3 (0.1)	0 (0)	0 (0)	
Endocrine therapy					
No	950 (30.2)	859 (30.0)	58 (33.0)	33 (30.3)	0.722
Yes	2191 (69.7)	1997 (69.9)	118 (67.0)	76 (69.7)	
NA	3 (0.1)	3 (0.1)	0 (0)	0 (0)	
Targeted therapy					
No	2504 (79.6)	2267 (79.3)	141 (80.1)	96 (88.1)	0.085
Yes	637 (20.3)	589 (20.6)	35 (19.9)	13 (11.9)	
NA	3 (0.1)	3 (0.1)	0 (0)	0 (0)	
Prolonged TTC^a^					
No	1807 (86.5)	1658 (86.5)	91 (82.7)	58 (90.6)	0.120
Yes	283 (13.5)	258 (13.5)	19 (17.3)	6 (9.4)	
Prolonged TTR^b^					
No	1095 (85.5)	106 (85.2)	53 (88.3)	36 (85.5)	0.567
Yes	186 (14.5)	175 (14.8)	7 (11.7)	4 (10.0)	

^a^Only those patients receiving chemotherapy with date information were included into analysis.

^b^Only those patients receiving radiotherapy with date information were included into analysis.

#### Disease outcome

The follow-up ranged from 1 to 128 months, with median follow-up duration of 52 months and 247 BC-related events. The estimated 5-year BCFI rate was 91.4%, and univariate analyses of the BCFI by prognostic factors are presented in Table 3. Patients’ clinicopathological characteristics, such as tumor size, axillary node status, tumor stage, histological grade, LVI, ER status, PR status, and molecular subtype, were all significantly correlated with the BCFI (p < 0.05), and age was marginally significant (p = 0.053) (Fig. S4). Regarding adjuvant treatment, chemotherapy and radiotherapy had a significant association with the BCFI (chemotherapy p = 0.001; radiation therapy p = 0.002).

**Table 3:** Univariate analysis of prognostic factors affecting BCFI and OS, The Ruijin cohort.

Characteristics	*P* value	
	BCFI	OS
Age (< 40 vs. 40–49 vs.50–70 vs. > 70)	0.053	0.001
Menstrual status (Pre/Peri- vs. Post-)	0.335	0.016
Residence (Large metropolitan vs. Metropolitan vs. Urban vs. Rural)	0.596	0.875
CCI (0 vs. 1–2 vs. 3 +)	0.173	0.756
Education level (Post-graduated vs. Degree vs. High school vs. Middle school)	0.062	0.127
BMI (< 25 vs. 25–30 vs. ≥ 30)	0.389	0.220
Histology type (IDC vs. Non-IDC)	0.304	0.695
Tumor size (≤ 2.0 cm vs. > 2.0 cm)	< 0.001	< 0.001
ALN status (Negative vs. Positive)	< 0.001	< 0.001
Tumor stage (I vs. II vs. III)	< 0.001	< 0.001
Histological grade (I vs. II vs. III)	< 0.001	0.050
LVI (Negative vs. Positive)	< 0.001	0.003
ER (Negative vs. Positive)	< 0.001	< 0.001
PR (Negative vs. Positive)	< 0.001	< 0.001
HER2 (Negative vs. Positive)	0.089	0.987
Ki67 (< 14% vs. ≥ 14%)	< 0.001	0.001
Molecular subtype (HR + /HER2- vs. HER2 + vs. TNBC)	0.001	0.001
Chemotherapy (No vs. Yes)	0.001	0.912
Radiation therapy (No vs. Yes)	0.002	0.177
Time to surgery (≤ 1w vs. 1–2 w vs. > 2w)	0.088	0.002

There were 126 deaths during the study period, with the estimated 5-year OS rate of 95.2%. The univariate analyses show that OS rate was significantly different among different age groups, as well as tumor size, axillary node status, tumor stage, histological grade, LVI, ER status, PR status and molecular subtype (p < 0.05) (Table 3). In contrast, comorbid conditions, residence areas, education levels, chemotherapy and radiotherapy had no significant association with the OS (P > 0.05).

#### Association between TTS and prognosis

**Figure 2:**
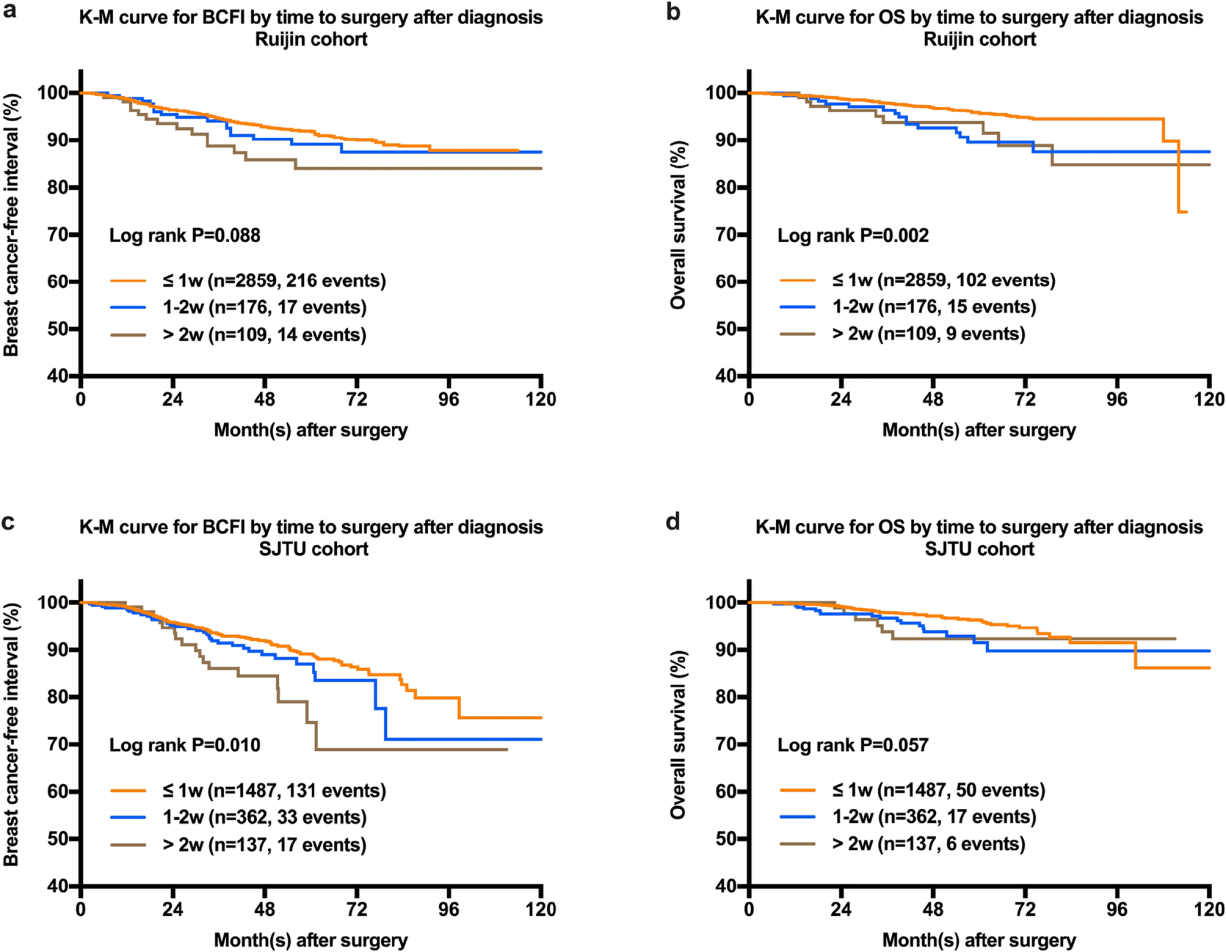
Kaplan–Meier curves of BCFI and OS in whole population by time to surgery after diagnosis. (**a**) The estimated 5-year BCFI rates in the Ruijin cohort for the ≤ 1w, 1-2w and > 2w groups were 91.8%, 89.2%, and 84.0%, respectively (*P* = 0.088). The *P* value for the ≤ 1w vs. 1-2w group was 0.408, for the 1-2w vs. > 2w group was 0.300, for the ≤ 1w vs. > 2w group was 0.036. (**b**) The estimated 5-year OS rates in the Ruijin cohort for the ≤ 1w, 1-2w and > 2w groups were 95.6%, 89.6% and 91.5%, respectively (*P* = 0.002). The *P* value for the ≤ 1w vs. 1-2w group was 0.004, for the 1-2w vs. > 2w group was 0.917, for the ≤ 1w vs. > 2w group was 0.021. (**c**) The estimated 5-year BCFI rates in the SJTU cohort for the ≤ 1w, 1-2w and > 2w groups were 89.2%, 87.0%, and 74.7%, respectively (*P* = 0.001). The *P* value for the ≤ 1w vs. 1-2w group was 0.174, for the 1-2w vs. > 2w group was 0.128, for the ≤ 1w vs. > 2w group was 0.004. (**d**) The estimated 5-year OS rates in the SJTU cohort for the ≤ 1w, 1-2w and > 2w groups were 96.4%, 91.5% and 92.4%, respectively (*P* = 0.057). The *P* value for the ≤ 1w vs. 1-2w group was 0.034, for the 1-2w vs. > 2w group was 0.901, for the ≤ 1w vs. > 2w group was 0.131.

Regarding different TTS groups, the estimated 5-year BCFI rates for ≤ 1 week, 1–2 weeks, and > 2 weeks groups were 91.8%, 87.5%, and 84.0%, with a marginal p value of 0.088 in the univariable model (Table 3, Fig. 2a). The estimated 5-year OS rates for ≤ 1 week, 1–2 weeks, and > 2 weeks groups were 95.6%, 89.6% and 91.5%, respectively (P = 0.002) (Table 3, Fig. 2b).

The multivariate analysis, which including the prognostic factors with significant or marginal p values in the univariate analysis, is presented in Table 4. Concerning the BCFI, tumor stage (P < 0.001), molecular subtype (P < 0.001), Ki67 index (P = 0.045), and TTS (P = 0.024) were found to be independent prognostic factors. Patients receiving surgery > 2 weeks had a significantly shorter BCFI than patients receiving surgery within 1 week (HR 1.80, 95% CI = 1.05–3.11, P = 0.034).

**Table 4:** Multivariate Cox proportional regression analysis of prognostic factors affecting BCFI and OS, The Ruijin cohort.

Characteristics	BCFI		OS	
	*HR* (95% CI)	*P* value	*HR* (95% CI)	*P* value
Age		0.160		**0.019**
< 40	1.00		1.00	
40–49	0.66 (0.43–1.03)	0.067	0.85 (0.40–1.79)	0.661
50–70	0.66 (0.44–0.98)	0.041	0.97 (0.49–1.93)	0.933
> 70	0.82 (0.47–1.44)	0.484	2.06 (0.93–4.59)	0.076
Menstrual status				**0.026**
Pre/Peri-	/		1.00	
Post-	/		2.38 (1.11–5.12)	
CCI		0.616		0.903
0	1.00		1.00	
1–2	0.88 (0.65–1.19)	0.400	0.91 (0.60–1.39)	0.668
3 +	0.83 (0.50–1.38)	0.473	0.92 (0.49–1.70)	0.783
Tumor stage^a^		** < 0.001**		** < 0.001**
I	1.00		1.00	
II	2.07 (1.44–2.96)	< 0.001	1.90 (1.14–3.15)	0.013
III	4.35 (2.86–6.61)	< 0.001	5.95 (3.35–10.56)	< 0.001
Histological grade		0.128		0.360
I	1.00		1.00	
II	8.90 (1.23–64.36)	0.030	1.26 (0.44–3.56)	0.670
III	11.21 (1.36–72.71)	0.024	1.76 (0.60–5.11)	0.302
NA	8.52 (1.06–59.70)	0.044	1.29 (0.41–4.06)	0.664
LVI		0.157		0.386
Negative	1.00		1.00	
Positive	1.31 (0.90–1.89)		1.27 (0.74–2.17)	
Molecular subtype^b^		**0.025**		**0.004**
HR + /HER2−	1.00		1.00	
HER2 +	1.22 (0.90–1.65)	0.197	1.21 (0.76–1.91)	0.424
TNBC	1.63 (1.14–2.32)	0.007	2.17 (1.36–3.45)	< 0.001
Ki67		**0.046**		**0.039**
< 14%	1.00		1.00	
≥ 14%	1.44 (1.01–2.07)		1.72 (1.03–2.88)	
Chemotherapy		0.971		0.110
No	1.00		1.00	
Yes	1.01 (0.69–1.47)		0.66 (0.39–1.10)	
Radiation therapy		**0.062**		0.902
No	1.00		1.00	
Yes	1.05 (0.78–1.41)		0.97 (0.64–1.48)	
Time to surgery		**0.023**		**0.008**
≤ 1 week	1.00		1.00	
1–2 weeks	1.23 (0.75–2.02)	0.406	2.05 (1.18–3.57)	0.011
> 2 weeks	1.77 (1.03–3.04)	0.040	2.09 (1.04–4.19)	0.037

Significant values are in bold.

^a^Cause tumor size and ALN status are components of tumor stage, we included tumor stage as an integral factor into multivariate analysis.

^b^Cause ER, PR and HER2 are components of molecular subtype, we included subtype as an integral factor into multivariate analysis.

Regarding OS, patient age (P = 0.002), menstrual status (P = 0.021), tumor stage (P < 0.001), molecular subtype (P < 0.001), Ki67 index (P = 0.003), and TTS (P = 0.001) were independently impact patient’s OS. Compared to patients receiving surgery within 1 week, both 1–2 week group (HR 2.17, 95% CI 1.24–3.78, P = 0.006) and > 2 week group (HR 2.07, 95% CI 1.04–4.13, P = 0.038) had a higher risk of death.

#### Association between TTS and BCFI according to clincopatholigcal characteristics

To further identify which patient population with a prolonged TTS had the worst BC-related survival, subgroup analyses including prognostic factors with significant or marginal p values in the multivariable model were performed (Table 5). The interaction p between molecular subtype (P < 0.001), tumor stage (P < 0.001), radiation (P = 0.003) and TTS reached statistical significance. In contrast, the interaction p between age and TTS did not reach statistical significance (P = 0.875).

**Table 5:** Exploratory analyses of BCFI rates by time to surgery after diagnosis according to patient characteristics and tumor subtype, The Ruijin cohort.

Characteristics	≤ 1week	1–2 weeks	> 2 weeks	*P* value
	Reference	HR (95% CI)	HR (95% CI)	
Age				
< 40	1	1.97 (0.75–5.18)	3.22 (1.12–9.33)*	0.057
40–49	1	0.66 (0.21–2.13)	1.61 (0.50–5.18)	0.554
50–70	1	0.72 (0.27–1.95)	1.47 (0.60–3.58)	0.562
> 70	1	0.30 (1.10–7.67)	1.27 (0.30–5.37)	0.096
P_interaction_	0.875			
Molecular subtype				
HR + /HER2−	1	0.86 (0.38–1.95)	1.35 (0.60–3.08)	0.711
HER2 +	1	1.43 (0.62–3.31)	3.66 (1.47–9.12)*	0.017
TNBC	1	1.82 (0.72–4.58)	1.42 (0.44–4.58)	0.399
P_interaction_	< 0.001			
Tumor stage				
Stage I	1	2.45 (0.96–6.28)	2.88 (1.05–9.38)*	0.040
Stage II	1	0.95 (0.44–2.03)	1.91 (0.97–3.76)	0.170
Stage III	1	1.13 (0.46–2.81)	0.97 (0.24–3.97)	0.963
P_interaction_	< 0.001			
Radiation therapy				
No	1	1.73 (0.87–3.45)	2.51 (1.21–5.19)*	0.019
Yes	1	0.93 (0.45–1.89)	1.30 (0.57–2.94)	0.799
P_interaction_	0.003			

*p < 0.05 compared with ≤ 1week group.

**Figure 3:**
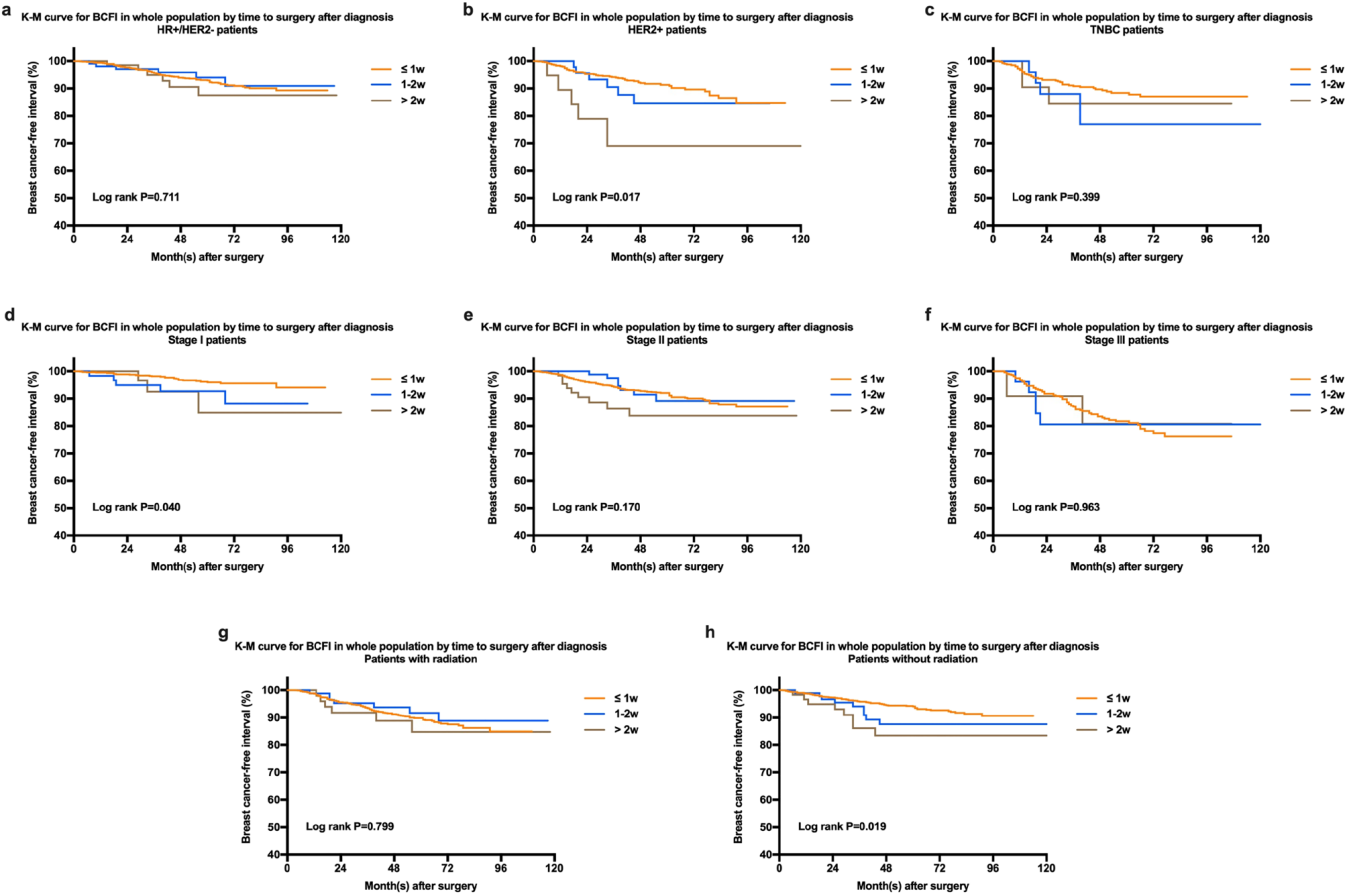
Kaplan–Meier curves of BCFI in different subgroup by time to surgery after diagnosis, the Ruijin cohort. (**a–c**) The estimated 5-year BCFI rates for HR + /HER2 −, HER2 + and TNBC patients by time to surgery after diagnosis. (**d–f**) The estimated 5-year BCFI rates for the Stage I, II and III groups by time to surgery after diagnosis. (**g,h**) The estimated 5-year BCFI rates for patients with or without radiation by time to surgery after diagnosis.

Among different subtypes, patients with HER2 disease (HR 3.66, 95% CI 1.47–9.12, P = 0.001) had a significantly poor BCFI rate when having more than 2 weeks prolonged interval between diagnosis and surgery (Fig. 3). Similarly, patient with stage I tumors, and without radiation had a significantly poor BCFI rate when having TTS > 2 weeks (Table 5, Fig. 3).

### The SJTU cohort

#### Patient characteristics

Besides BC patients of Ruijin Hospital, a total of 1986 patients were included from SJTU-BCDB database, and identified as the SJTU cohort. The median time from BC diagnosis to surgery was 4 days (range from 0 to 89 days). Patients who received surgery within ≤ 1 week, 1–2 weeks, and > 2 weeks after diagnosis accounted for 74.9%, 18.2%, and 6.9% of all the patients, respectively (Fig. S2b). Descriptive results according to TTS are detailed in Table 6.

**Table 6:** Baseline clinical and pathological characteristics by time to surgery after diagnosis, The SJTU cohort.

Characteristics	Total N = 1986 (%)	≤ 1week N = 1487 (%)	1–2 weeks N = 362 (%)	> 2 weeks N = 137 (%)	*P* value
Age (y/o)					
< 40	267 (13.4)	177 (11.9)	57 (15.7)	33 (24.1)	< 0.001
40–49	706 (35.5)	547 (36.8)	119 (32.9)	40 (29.2)	
50–70	914 (46.0)	697 (46.9)	157 (43.4)	60 (43.8)	
> 70	99 (5.0)	66 (4.4)	29 (8.0)	4 (2.9)	
Menstrual status					
Pre/Peri-	1111 (55.9)	828 (55.7)	199 (55.0)	84 (61.3)	0.410
Post-	875 (44.1)	659 (44.3)	163 (45.0)	53 (38.7)	
CCI					
0	1444 (72.7)	1093 (73.5)	249 (68.8)	102 (74.5)	0.029
1–2	414 (20.8)	311 (20.9)	82 (22.7)	21 (15.3)	
3 +	128 (6.4)	83 (5.6)	31 (8.6)	14 (10.2)	
Breast surgery					
BCS	586 (29.5)	457 (30.7)	97 (26.8)	32 (23.4)	0.089
Mastectomy	1400 (70.5)	1030 (69.3)	265 (73.2)	105 (76.7)	
Axillary surgery					
SLNB	956 (48.1)	745 (50.1)	160 (44.2)	51 (37.2)	0.022
ALND	1014 (51.1)	731 (49.2)	198 (54.7)	85 (62.0)	
NA	16 (0.8)	11 (0.7)	4 (1.1)	1 (0.7)	
Histology type					
IDC	1775 (89.4)	1341 (90.2)	317 (87.6)	117 (85.4)	0.103
Non-IDC	211 (10.6)	146 (9.8)	45 (12.4)	20 (14.6)	
Tumor size					
≤ 2.0 cm	960 (48.3)	729 (49.0)	169 (46.7)	62 (45.3)	0.549
> 2.0 cm	1026 (51.7)	758 (51.0)	193 (53.3)	75 (54.7)	
ALN status					
Negative	1114 (56.1)	841 (56.6)	204 (56.4)	69 (50.4)	0.374
Positive	872 (43.9)	646 (43.4)	158 (43.6)	68 (49.6)	
Tumor stage					
I	679 (34.2)	526 (35.4)	117 (32.3)	36 (26.3)	0.110
II	966 (48.6)	700 (47.1)	189 (52.2)	77 (56.2)	
III	341 (17.2)	261 (17.6)	56 (15.5)	24 (17.5)	
Histological grade					
I	125 (6.3)	95 (6.4)	21 (5.8)	9 (6.6)	0.845
II	1276 (64.2)	962 (64.7)	231 (63.8)	83 (60.6)	
III	368 (18.5)	276 (18.6)	64 (17.7)	28 (20.4)	
NA	217 (10.9)	154 (10.4)	46 (12.7)	17 (12.4)	
ER					
Negative	464 (23.4)	343 (23.1)	83 (22.9)	38 (27.7)	0.455
Positive	1522 (76.6)	1144 (76.9)	279 (77.1)	99 (72.3)	
PR					
Negative	581 (29.3)	426 (28.6)	113 (31.2)	42 (30.7)	0.587
Positive	1405 (70.7)	1061 (71.4)	249 (68.8)	95 (69.3)	
HER2					
Negative	1501 (75.6)	1131 (76.1)	271 (74.9)	99 (72.3)	0.576
Positive	485 (24.4)	356 (23.9)	91 (25.1)	38 (27.7)	
Ki67					
< 14%	485 (24.4)	364 (24.5)	84 (23.2)	37 (27.0)	0.674
≥ 14%	1501 (75.6)	1123 (75.5)	278 (76.8)	100 (73.0)	
Molecular subtype					
HR + /HER2 −	1245 (62.7)	935 (62.9)	229 (63.3)	81 (59.1)	0.789
HER2 +	485 (24.4)	356 (23.9)	91 (25.1)	38 (27.7)	
TNBC	256 (12.9)	196 (13.2)	42 (11.6)	18 (13.1)	

#### Disease outcome

The follow-up ranged from 1 to 137 months, with median follow-up duration of 58 months, and 126 BC-related events. The estimated 5-year BCFI rate was 88.1%, and univariate analyses of the BCFI by prognostic factors are presented in Table 7. Age, comorbid conditions, tumor size, axillary node status, tumor stage, histological grade, LVI, ER status, PR status, and molecular subtype, were all significantly correlated with the BCFI (p < 0.05).

**Table 7:** Univariate analysis of prognostic factors affecting BCFI and OS, The SJTU cohort.

Characteristics	*P* value	
	BCFI	OS
Age (< 40 vs. 40–49 vs.50–70 vs. > 70)	< 0.001	< 0.001
Menstrual status (Pre/Peri- vs. Post-)	0.144	< 0.001
CCI (0 vs. 1–2 vs. 3 +)	0.031	< 0.001
Histology type (IDC vs. Non-IDC)	0.998	0.521
Tumor size (≤ 2.0 cm vs. > 2.0 cm)	< 0.001	0.019
ALN status (Negative vs. Positive)	< 0.001	< 0.001
Tumor stage (I vs. II vs. III)	< 0.001	< 0.001
Histological grade (I vs. II vs. III)	0.001	0.003
ER (Negative vs. Positive)	< 0.001	< 0.001
PR (Negative vs. Positive)	< 0.001	< 0.001
HER2 (Negative vs. Positive)	0.326	0.987
Ki67 (< 14% vs. ≥ 14%)	0.004	0.020
Molecular subtype (HR + /HER2- vs. HER2 + vs. TNBC)	< 0.001	< 0.001
Chemotherapy (No vs. Yes)	0.341	0.353
Radiation therapy (No vs. Yes)	0.174	0.475
Time to surgery (≤ 1w vs. 1–2 w vs. > 2w)	0.010	0.057

There were 73 deaths during the study period, with the estimated 5-year OS rate of 95.4%. The univariate analyses show that OS rate was significant among the different age groups, as well as menstrual status, CCI, tumor size, axillary node status, tumor stage, histological grade, LVI, ER status, PR status and molecular subtype (p < 0.05) (Table 7).

#### Association between TTS and prognosis

Among the SJTU cohort, the estimated 5-year BCFI rates for the ≤ 1 week, 1–2 weeks, and > 2 weeks groups were 89.2%, 87.0%, and 74.7%, with a significantly p value of 0.010 among the three groups in the univariate model (Table 7, Fig. 2c). The estimated 5-year OS rates for the ≤ 1 week, 1–2 weeks, and > 2 weeks groups were 96.4%, 91.5% and 92.4%, respectively (P = 0.057) (Table 7, Fig. 2d).

#### Exploratory joint analysis combining Ruijin cohort with SJTU cohort of prolonged TTS and prognosis

There were totally 5130 patients when combining Ruijin cohort with SJTU cohort, containing 4346 patients with TTS ≤ 1 week, 538 patients with 1–2 weeks, and 246 patients > 2 weeks (Table 8). In a joint analysis, among those 5130 patients, the estimated 5-year BCFI rates for the ≤ 1 week, 1–2 weeks, and > 2 weeks groups were 91.0%, 87.9%, and 78.9%, and the estimated 5-year OS rates for the ≤ 1 week, 1–2 weeks, and > 2 weeks groups were 95.8%, 90.6%, and 91.5%, both reaching the significantly p value (p < 0.001) among the three groups (Fig. S5a, b).

**Table 8:** Clinicopathological characteristics of the Ruijin and SJTU cohorts’ combination by time to surgery after diagnosis.

Characteristics	Before propensity score matched (N = 5130)			*P* value	After propensity score matched (N = 1484)			*P* value
	≤ 1 week N = 4346 (%)	1–2 weeks N = 538 (%)	> 2 weeks N = 246 (%)		≤ 1 week N = 1060 (%)	1–2 weeks N = 212 (%)	> 2 weeks N = 212 (%)	
Age (y/o)								
< 40	413 (9.5)	85 (15.8)	46 (18.7)	** < 0.001**	175 (16.5)	37 (17.5)	40 (18.9)	0.403
40–49	1189 (27.4)	172 (32.0)	64 (26.0)		365 (34.4)	72 (34.0)	57 (26.9)	
50–70	2344 (53.9)	226 (42.0)	109 (44.3)		439 (41.4)	85 (40.1)	92 (43.4)	
> 70	400 (9.2)	55 (10.2)	27 (11.0)		81 (7.6)	18 (8.5)	23 (10.8)	
CCI								
0	2794 (64.3)	360 (66.9)	145 (58.9)	**0.003**	659 (62.2)	130 (61.3)	128 (60.4)	0.322
1–2	1189 (27.4)	128 (23.8)	64 (26.0)		287 (27.1)	54 (25.5)	51 (24.1)	
3 +	363 (8.4)	50 (9.3)	37 (15.0)		114 (10.8)	28 (13.2)	33 (15.6)	
Breast surgery								
BCS	1250 (28.8)	143 (26.6)	57 (23.2)	0.109	314 (29.6)	57 (26.9)	50 (23.6)	0.179
Mastectomy	3096 (71.2)	395 (73.4)	189 (76.8)		746 (70.4)	155 (73.1)	162 (76.4)	
Axillary surgery								
SLNB	2317 (53.3)	252 (46.9)	114 (46.3)	**0.003**	539 (50.8)	100 (47.2)	98 (46.2)	0.107
ALND	2010 (46.3)	280 (52.0)	129 (52.4)		519 (49.0)	109 (51.4)	111 (52.4)	
NA	19 (0.4)	6 (1.1)	3 (1.2)		2 (0.2)	3 (1.4)	3 (1.4)	
Histology type								
IDC	3852 (88.6)	475 (88.3)	214 (87.0)	0.772	1007 (95.0)	197 (92.9)	195 (92.0)	0.149
Non-IDC	494 (11.4)	63 (11.7)	32 (13.0)		53 (5.0)	15 (7.1)	17 (8.0)	
Tumor stage								
I	1567 (36.1)	176 (32.7)	70 (28.5)	**0.026**	279 (26.3)	55 (25.9)	56 (26.4)	0.862
II	2084 (48.0)	280 (52.0)	141 (57.3)		594 (56.0)	122 (57.5)	125 (59.0)	
III	695 (16.0)	82 (15.2)	35 (14.2)		187 (17.7)	35 (16.5)	31 (14.6)	
Histological grade								
I	237 (5.5)	31 (5.8)	21 (8.5)	**0.016**	84 (7.9)	15 (7.1)	21 (17.5)	0.272
II	2375 (54.6)	320 (59.5)	130 (52.8)		727 (68.6)	144 (67.9)	129 (60.8)	
III	1226 (29.1)	122 (22.7)	63 (25.6)		249 (23.5)	53 (25.0)	62 (29.2)	
NA	468 (10.8)	65 (12.1)	32 (13.0)		0 (0.0	0 (0.0)	0 (0.0)	
Molecular subtype								
HR + /HER2 −	2638 (60.7)	334 (62.1)	150 (61.0)	0.719	655 (61.8)	125 (59.0)	131 (61.8)	0.731
HER2 +	1103 (25.4)	137 (25.5)	57 (23.2)		262 (24.7)	59 (27.8)	48 (22.6)	
TNBC	605 (13.9)	67 (12.5)	39 (15.9)		143 (13.5)	28 (13.2)	33 (15.6)	

Significant values are in bold.

When analyzed by exploratively four grouping as < 1 week, 1–2 weeks, 2–4 weeks, and > 4 weeks, the 246 patients of > 2 weeks groups were separated as 197 patients with 2–4 weeks and 49 patients > 4 week. The estimated 5-year BCFI rates for the ≤ 1 week, 1–2 weeks, 2–4 weeks, and > 4 weeks groups were 91.0%, 87.9%, 79.6%, and 76.3%, and the estimated 5-year OS rates for the ≤ 1 week, 1–2 weeks, 2–4 weeks, and > 4 weeks groups were 95.8%, 90.6%, 91.8% and 90.2%, both with a significantly p value (p = 0.001) among the four groups (Fig. S5c,d).

We chose the combination of Ruijin & SJTU cohort to perform PSM analysis. After matching based on the propensity score, 1060 patients with TTS ≤ 1 week, 212 patients with 1–2 weeks, and 212 patients with TTS > 2 weeks were identified (Table 8). The baseline characteristics including age, CCI, tumor stage, molecular subtype, pathological type, tumor grade and surgery type were comparable after PSM (p > 0.05). Among those 1484 patients after PSM, the estimated 5-year BCFI rates for the ≤ 1 week, 1–2 weeks, and > 2 weeks groups were 91.0%, 88.0%, and 81.5% (p = 0.008), and the estimated 5-year OS rates for the ≤ 1 week, 1–2 weeks, and > 2 weeks groups were 96.2%, 88.1%, and 91.3% (p < 0.001), which had significant disease outcome difference (Fig. S6a,b).

## Discussion

The hypothesis exists that prolonged interval from pathological biopsy to surgery might allow BC cells to proliferate and spread to other sites, which causes the impaired prognosis^17^. However, there has been little consensus about the relationship between prolonged surgical initiation and BC patient survival, especially in the era of modern treatment of BC. To our knowledge, the present study has both the largest single institute cohort (Ruijin cohort) and multicenter cohort (Ruijin cohort *plus* SJTU cohort) to examine the association between prolonged time to surgery and early stage breast cancer prognosis in modern era of adjuvant treatment. We found that long interval from biopsy to definite surgery is associated with worse BCFI and OS, providing evidence that patients with a prolonged TTS (> 2 weeks) after BC diagnosis may experience poorer survival than patients who undergo surgery with short TTS. A consistent trend between greatly long interval (e.g. > 4 weeks) and inferior survival was also observed in our study. According to these results, the efforts to shorten TTS for BC patients are extremely necessary. Furthermore, we found that several factors, including age and comorbid conditions, are correlated to prolonged interval to BC surgery, and the elevated recurrence risk associated with prolonged TTS may vary by the tumor subtype, tumor stage, and radiation treatment.

Concerning the factors related to prolonged TTS, time to surgery is affected by the cancer care pathway from diagnosis to making appointment(s) and treatment. In clinical scenario, an adequate time is needed for treatment planning before definite surgery, such as pathology and imaging assessments^18–21^. Besides this, the time spent waiting for receptor testing and considering neoadjuvant chemotherapy to shrink operable tumors would also prolong TTS. In addition, the preoperative genetic testing or planning for oncoplastic surgery may also largely contribute to long interval between pathological biopsy and surgery^22^. Regarding patients’ factors, patients may also prolong decision making by seeking multiple opinions or request delays to accommodate their work or personal schedules. Other factors, such as patient’s anxiety, age, comorbid conditions, and some sociodemographic factors (e.g. patient’s education level and residence area), can also confer additional delays^23–27^. Furthermore, since 2020, challenges from the COVID-19 pandemic, including the risk of patient and staff exposure to SARS-CoV-2 and the need for personal protective equipment, ventilators, and medical staff who could otherwise be deployed to care for patients with COVID-19, would also prolong TTS^28,29^.

Our study found that long interval to surgery would impair patient’s disease outcome, which was consistent with other studies. Eaglehouse et al. reported a significantly increased risk (30%) of all-cause death with a TTS ≥ 36 days compared to 1–21 days among BC patients in the universal-access U.S. Military Health System^7^. Similarly, they found that an increased risk of mortality associated with TTS ≥ 36 days tended to be consistent when analyzed by tumor stage. Another study from Smith et al. focused on young BC patients (aged from 15 to 39 years)^8^, which found that the 5-year OS rate in patients with TTS ≥ 6 weeks (80%) was significantly lower than that patients with TTS less than 2 weeks (90%). Moreover, Bleicher et al. found a significantly increased risk (10%) of all-cause mortality for each incremental 30-day interval between diagnosis and surgery and a 26% higher risk of BC-specific mortality for each 60-day increase in TTS^9^. Nevertheless, several studies have shown no association between a prolonged interval and survival^30,31^. The possible reasons for the inconsistency were varied, and the bias of information from the cancer registry database might be one possible reason. Most of the above studies used nationwide cancer registry data as a data source, which tend to have limited details and may be inaccurate. In contrast, our study used electronic medical record data derived from a single institution (Ruijin cohort), which would contain more accurate clinicopathological information and survival data. Moreover, those reports included patients from twenty years ago, which has not integrated modern era treatment advances, including adjuvant anti-HER2 therapy and ovarian function suppression treatment, but our study included patients in recent ten years with these systemic treatment advances.

Obviously, TTS reported in our study (median TTS: 4 days) was significantly shorter than that in the above-mentioned studies, indicating the difference of patient care system between China and America or other countries. As Fig. S1 illustrated, we have explained our diagnostic and surgical procedure in methods. In China, due to our large patient population, the cancer care system needs accelerating treatment procedure to shorten the hospitalization time, and patients are also willing to be diagnosed and treated within a relative short time period^5,10,11^. Moreover, due to the care system, treatment cost is much higher covered by the medical insurance if patients are treated in the ward, so most of patients will receive image assessments, core needle biopsy and following surgery in the same hospitalization period, leading to comparatively short waiting period between diagnosis and treatment. Therefore, to our knowledge, this study is also the first study to evaluate the impact of prolonged TTS at weekly length scale on BC patient prognosis, which is really hard to conduct in other countries.

Regarding the relation between prolonged TTS and poor survival, one of possible reason may be the increased Ki67 index after diagnostic biopsy. We previously reported a significantly higher Ki67 expression value in surgical samples than in CNB samples, and BC patients with longer surgery waiting times after biopsy had a higher chance of Ki67 increases, which was possibly due to wound healing and a stromal reaction^32^. Although the Ki67 index, as a BC proliferation biomarker, has not been confirmed to worsen the disease outcome, its increase might reflect tumor progression^33^. In the exploratory analysis of our manuscript, we observed the relationship between increase of Ki67 and longer TTS. Of course, there was a consistence debate about the Ki67 heterogeneity in CNB sample, which warrants further study. Another reason that may explain the poor disease outcome for patients with a prolonged TTS is the difference in tumor immune microenvironment. One former study from Mathenge EG et al. found that CNB created a distinctly immunosuppressive tumor microenvironment with a higher frequency of myeloid-derived suppressor cells (MDSCs) accompanied by reduced CD4 + T cells, CD8 + T cells, and macrophages^34^. Our team also reported that TILs were significantly higher in surgical samples than in CNB samples, and the increasing of TILs were associated with a longer TTS and a worse BCFI, especially in HER2 + patients, consistent with our finding^35,36^. In addition, Mathenge EG et al. found that, in the mouse model, the impact of CNB includes creation of a pro-metastatic tumor microenvironment with elevated TGF-β/SOX-4-associated epithelial-mesenchymal transition (EMT) and significantly higher circulating tumor cells (CTCs) levels^34^. Therefore, the association between longer TTS and survival is more likely due to tumor biological behavior change rather than sample loco-reginal spread phenomenon. One reasonable hypothesis might be that CNB stimulates tumor cells proliferation, destroys the barrier, creates an immunosuppressive tumor microenvironment, increases epithelial-mesenchymal transition (EMT), and facilitates release of CTCs during TTS, all of which likely contribute to the development of distant metastases, and worsen the prognosis. Potential reasons for prolonged TTS and its effects on survival is warranted to be better researched.

Regarding the interaction between tumor subtypes, TTS and prognosis, we firstly found patients with stage I TTS had a worse disease outcome when having prolonged TTS, possibly due to the relatively lower baseline recurrence risk in these patients compared to those patients with stage II or III disease. Moreover, the interaction of TTS and prognosis were also observed in HER2 positive patients and those not receive radiation treatment, indicating the possible higher proliferation activation after CNB in HER2 positive tumor and lacking of local control in patients who not treated with radiation. Interestingly, the results from several studies have provided evidence that a long interval from surgery to adjuvant chemotherapy^37^ or from chemotherapy to radiotherapy^38^ might cause a poor prognosis in certain populations. Taken together, our data support that care pathway for BC patients with short time period between biopsy and surgery is important. Neoadjuvant systemic therapy in lieu of surgery may be a reasonable option for these patients if they need a long waiting time period for surgery, such as the COVID-19 pandemic^39^. For example, one recent study from UK found a clinically significant impact on cancer survival if delays to the 2-week-wait cancer pathway are extensive and prolonged^40^. Therefore, some groups and scientific societies have made practical recommendations to try mitigating the deleterious effect of COVID-19 pandemic on cancer care. Our study will be helpful to possible BC patient selection, treatments and schedules tailored according to BC patients and tumor criteria^40,41^.

The retrospective analysis is a limitation of this study. However, it is difficult to conduct a randomized trial to investigate the optimal TTS. In addition, this study did not evaluate the time interval between symptom presentation and diagnosis. Finally, subgroup analysis was performed on a relatively small sample size, which was another limitation, we can’t conclude whether patients with longer surgery delay would be related with even worse disease outcome, or longer delay was positively linearly associated with worse disease outcome. Further research with more number of included patients is needed to evaluate the role of TTS in these subgroups of BC patients.

## Conclusions

Our study found that BC patients with elderly age and medical comorbidities were more likely to have a prolonged interval between diagnosis and surgery initiation. Prolonged time to surgery (more than 2 weeks) after BC diagnosis was associated with poor disease outcomes, which may vary by tumor stage, molecular subtype, and radiotherapy, indicating we need to shorten the time interval between initiate diagnosis and surgery, thus to improve BC survival.

## Supplementary Information

Supplementary Information 1.

Supplementary Information 2.

Supplementary Information 3.

Supplementary Information 4.

Supplementary Information 5.

Supplementary Information 6.

Supplementary Legends.

## Data Availability

The datasets used and/or analysed during the current study are available from the corresponding author on reasonable request.

## Abbreviations

ALN: Axillary lymph node
ALND: Axillary lymph node dissection
BC: Breast cancer
BCFI: Breast cancer-free interval
BCS: Breast-conserving surgery
CCI: Charlson Comorbidity index
CNB: Core needle biopsy
COVID-19: Coronavirus disease 2019
CTCs: Circulating tumor cells
EMT: Epithelial-mesenchymal transition
ER: Estrogen receptor
HER2: Human epidermal growth factor receptor 2
HR: Hormone receptor
IDC: Invasive breast cancer
LVI: Lymph-vascular invasion
MDSCs: Myeloid-derived suppressor cells
OS: Overall survival
PR: Progesterone receptor
PSM: Propensity score match
SJTU-BCDB: Shanghai Jiaotong University Breast Cancer Database
SLNB: Sentinel lymph node biopsy
TNBC: Triple negative breast cancer
TTC: Time to chemotherapy
TTR: Time to radiotherapy
TTS: Time to surgery
