# Supplementary figures and images for: Time interval between breast cancer diagnosis and surgery is associated with disease outcome
Source: Sci Rep. 2023 Jul 26;13:12091. doi: 10.1038/s41598-023-39259-3 (PMC10372101; doi:10.1038/s41598-023-39259-3)

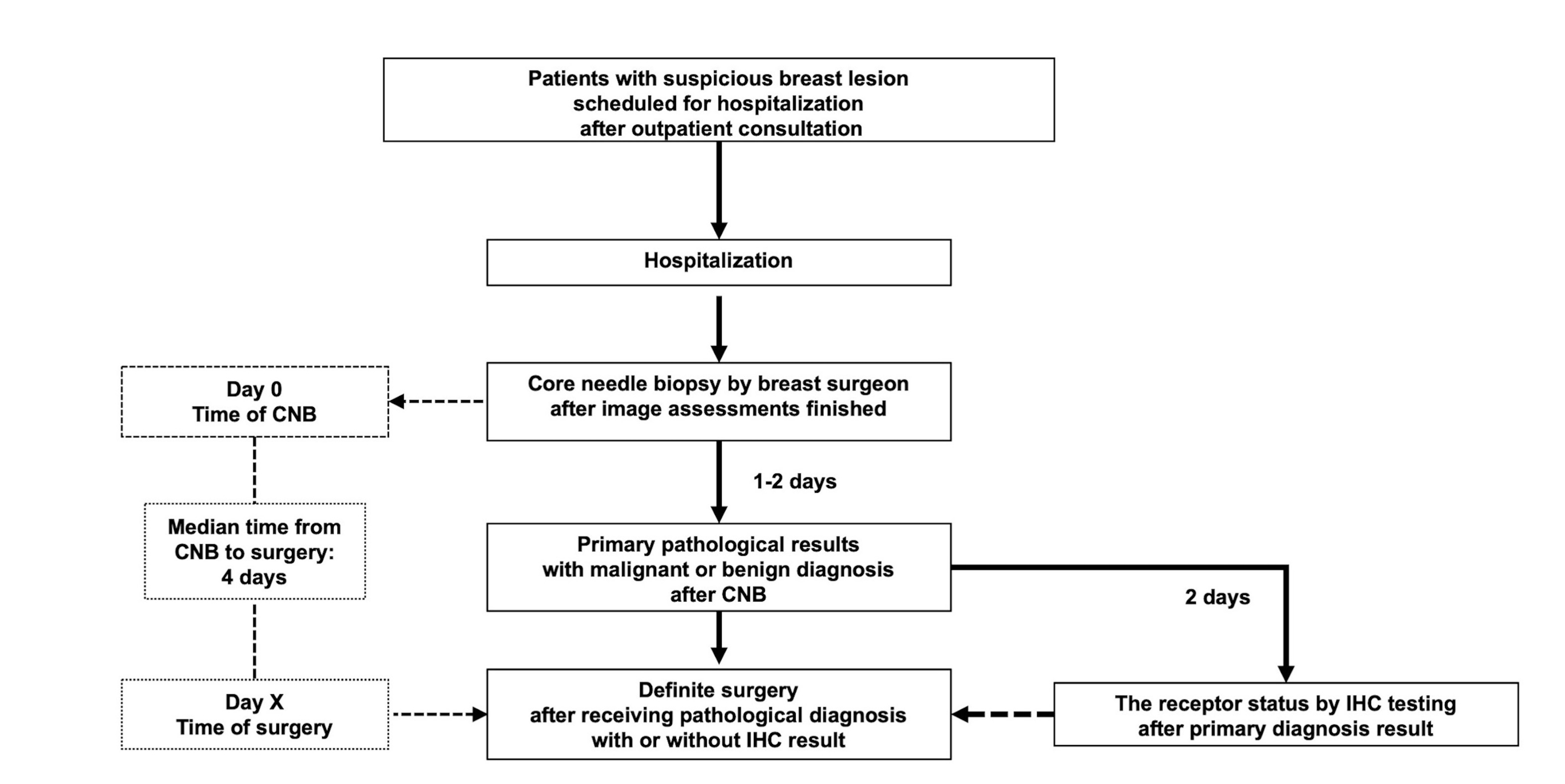

Supplement: Supplementary file 1 — Supplementary Information 1. [file 41598_2023_39259_MOESM1_ESM.jpg]

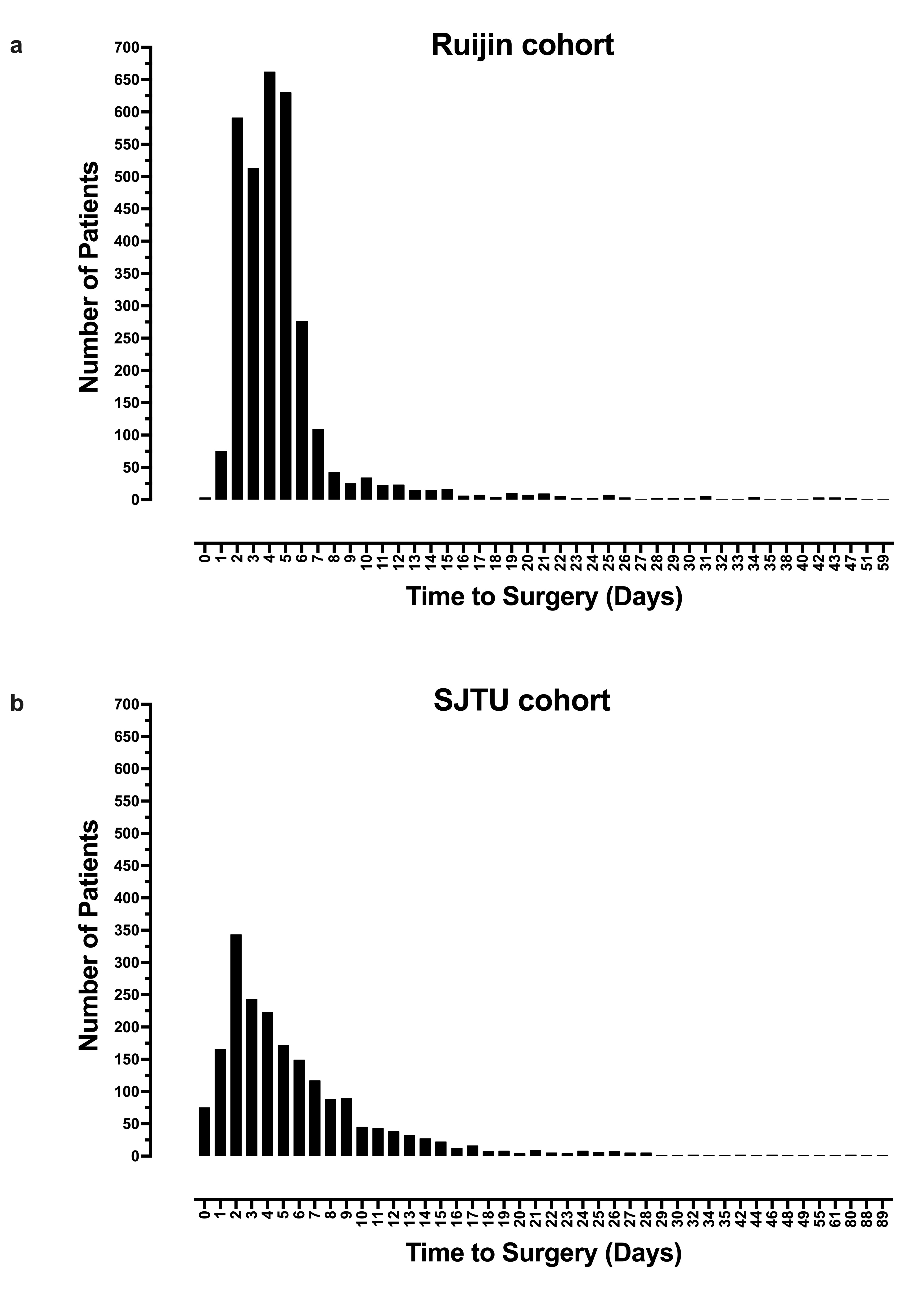

Supplement: Supplementary file 2 — Supplementary Information 2. [file 41598_2023_39259_MOESM2_ESM.jpg]

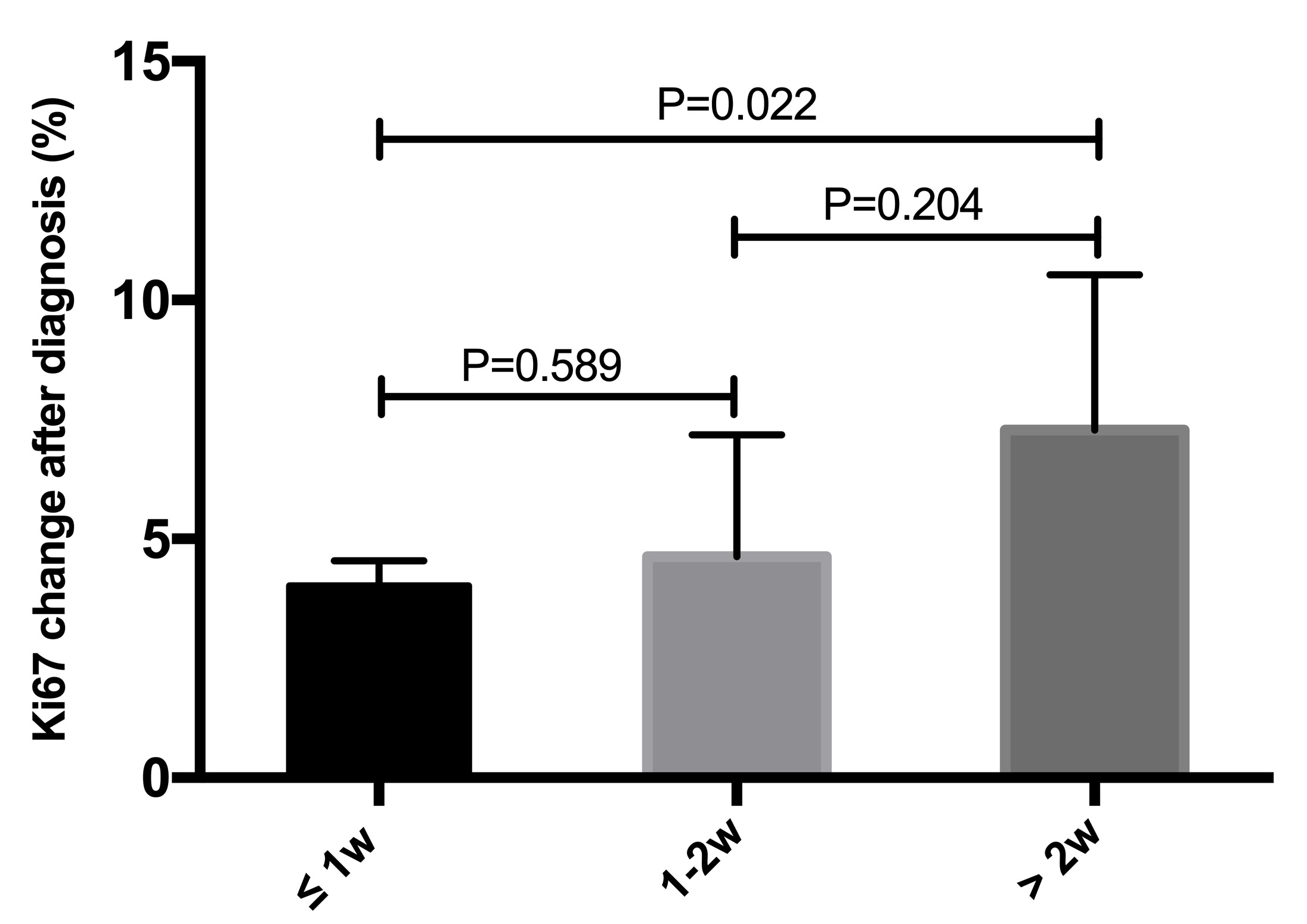

Supplement: Supplementary file 3 — Supplementary Information 3. [file 41598_2023_39259_MOESM3_ESM.jpg]

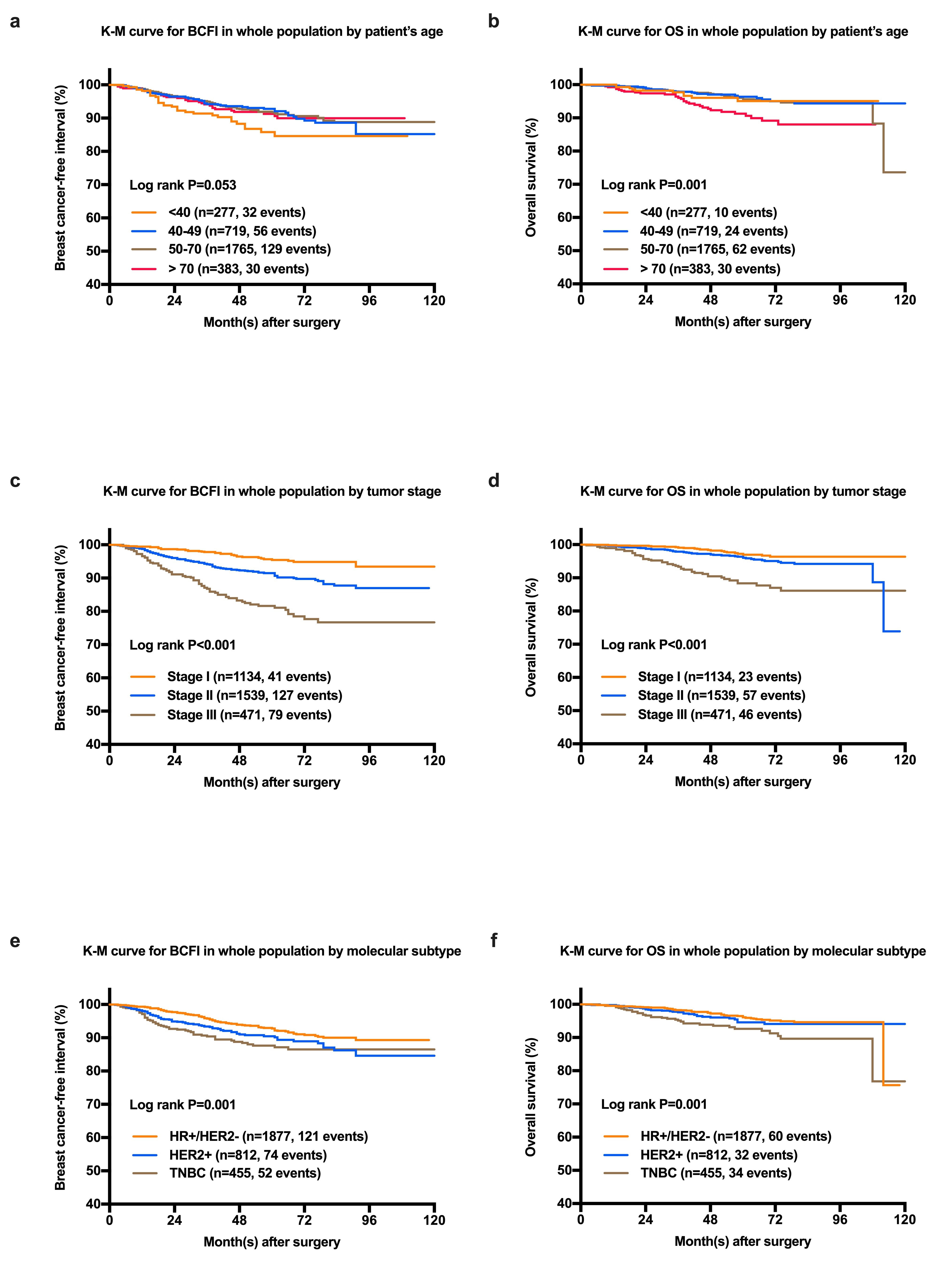

Supplement: Supplementary file 4 — Supplementary Information 4. [file 41598_2023_39259_MOESM4_ESM.jpg]

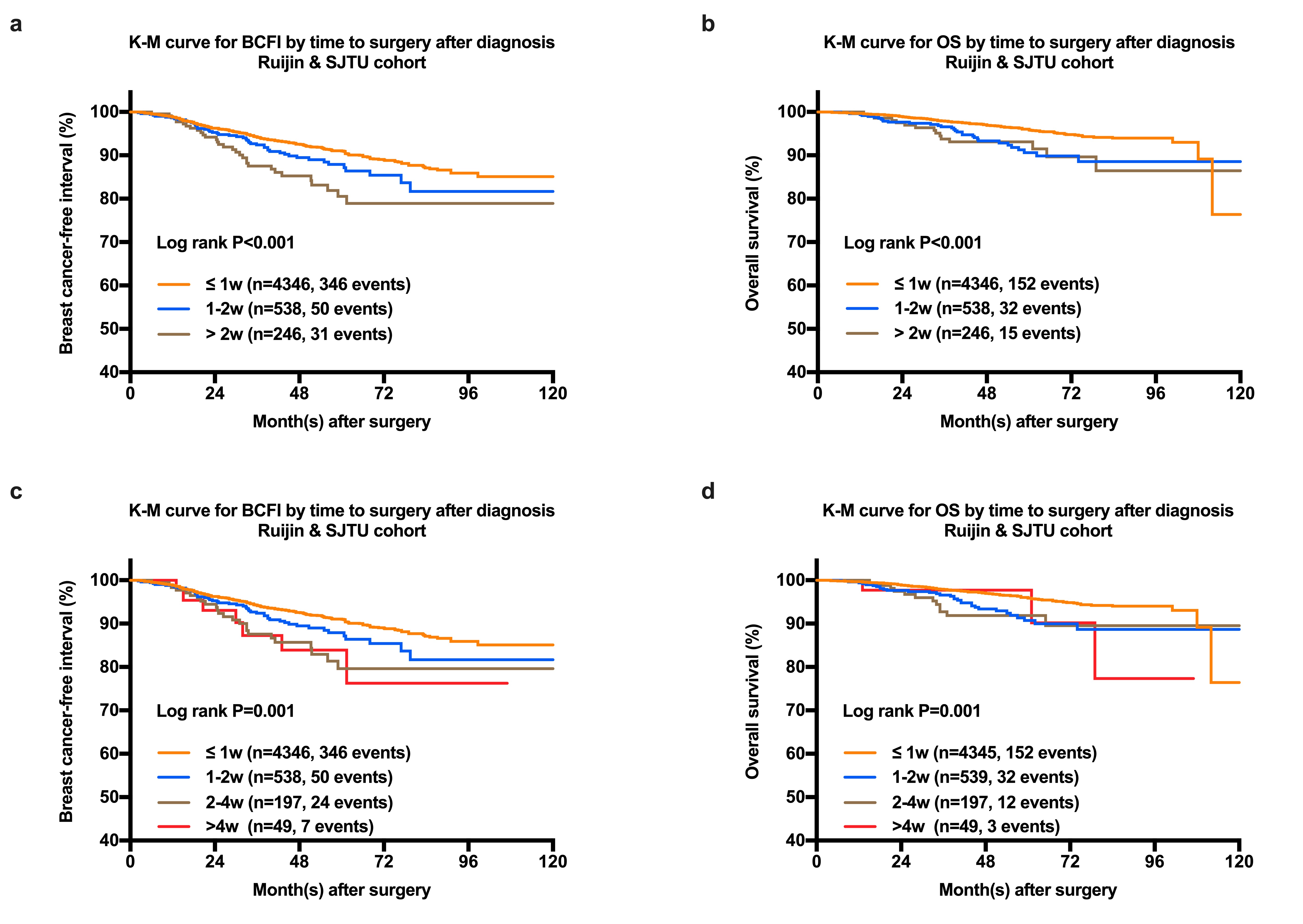

Supplement: Supplementary file 5 — Supplementary Information 5. [file 41598_2023_39259_MOESM5_ESM.jpg]

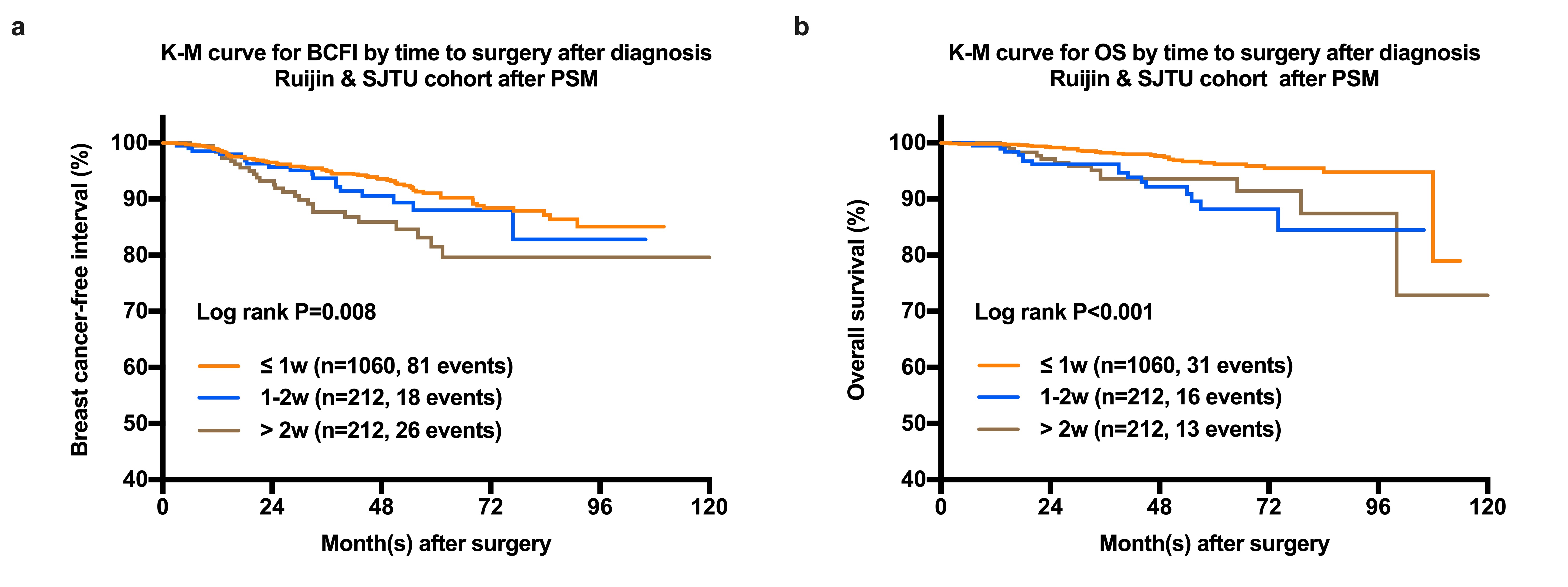

Supplement: Supplementary file 6 — Supplementary Information 6. [file 41598_2023_39259_MOESM6_ESM.jpg]
