# Supplementary material for: Time interval between breast cancer diagnosis and surgery is associated with disease outcome
Source: Sci Rep. 2023 Jul 26;13:12091. doi: 10.1038/s41598-023-39259-3 (PMC10372101; doi:10.1038/s41598-023-39259-3)
Supplement: Supplementary file 7 — Supplementary Legends. [file 41598_2023_39259_MOESM7_ESM.docx]

**Figure legends of supplementary figures**

**Figure S1** The diagnostic and surgical procedures of study population. The median time from BC diagnosis to surgery in this study was 4 days.

**Figure S2** Distribution of patients according to time from BC diagnosis to surgery. (a) The Ruijin cohort. (b) The SJTU cohort.

**Figure S3** Ki67 expression change between diagnosis and surgery at different surgery time intervals, the Ruijin cohort. The mean Ki67 expression change for the ≤ 1w, 1-2w and > 2w groups were 4.0%, 4.6% and 7.3%, respectively. The *P* values of T test were 0.589 for ≤ 1w vs. 1-2w, 0.204 for 1-2w vs. > 2w and 0.022 for ≤ 1w vs. >2w, respectively.

**Figure S4** Kaplan-Meier curves of BCFI and OS in whole population by different factors, the Ruijin cohort. (a) The estimated 5-year BCFI rates for the <40, 40-49, 50-70, and >70 groups were 85.8%, 92.6%, 91.8% and 91.3%, respectively (*P*= 0.053). (b) The estimated 5-year OS rates for the <40, 40-49, 50-70, and >70 groups were 95.1%, 96.4%, 95.6% and 91.3%, respectively (*P*= 0.001). (c) The estimated 5-year BCFI rates for the Stage I, II and III groups were 95.4%, 91.5%, and 81.6%, respectively (*P*< 0.001). (d) The estimated 5-year OS rates for the Stage I, II and III groups were 96.9%, 96.0% and 88.3%, respectively (*P*< 0.001). (e) The estimated 5-year BCFI rates for the HR+/HER2-, HER2+ and TNBC groups were 92.7%, 90.5%, and 87.6%, respectively (*P*= 0.001). (f) The estimated 5-year OS rates for the HR+/HER2-, HER2+ and TNBC groups were 96.1%, 94.6% and 92.6%, respectively (*P*= 0.001).

**Figure S5** Kaplan-Meier curves of BCFI and OS in the Ruijin & SJTU cohort by time to surgery after diagnosis. (a) The estimated 5-year BCFI rates in the Ruijin & SJTU cohort for the ≤ 1w, 1-2w, and > 2w groups were 91.0%, 87.9%, and 78.9%, respectively (*P*< 0.001). The P value for the ≤ 1w vs. 1-2w group was 0.043, for the 1-2w vs. >2w group was 0.102, for the ≤ 1w vs. >2w group was < 0.001. (b) The estimated 5-year OS rates in the Ruijin & SJTU cohort for the ≤ 1w, 1-2w, and > 2w groups were 95.8%, 90.6%, and 91.5%, respectively (*P*< 0.001). The P value for the ≤ 1w vs. 1-2w group was < 0.001, for the 1-2w vs. >2w group was 0.821, for the ≤ 1w vs. >2w group was 0.009. (c) The estimated 5-year BCFI rates in the Ruijin & SJTU cohort for the ≤ 1w, 1-2w, 2-4w, and > 4w groups were 91.0%, 87.9%, 79.6%, and 76.3%, respectively (P= 0.001). The P value for the ≤ 1w vs. 1-2w group was 0.043, for the ≤ 1w vs. 2-4w group was 0.002, for the ≤ 1w vs. >4w group was 0.057. (d) The estimated 5-year OS rates in the Ruijin & SJTU cohort for the ≤ 1w, 1-2w, 2-4w, and > 4w groups were 95.8%, 90.6%, 91.8% and 90.2%, respectively (P= 0.001). The P value for the ≤ 1w vs. 1-2w group was < 0.001, for the ≤ 1w vs. 2-4w group was 0.019, for the ≤ 1w vs. >4w group was 0.053.

**Figure S6** Kaplan-Meier curves of BCFI and OS in the Ruijin & SJTU cohort after PSM analysis. (a) The estimated 5-year BCFI rates in the Ruijin & SJTU cohort after PSM analysis for the ≤ 1w, 1-2w, and > 2w groups were 91.0%, 88.0%, and 81.5%, respectively (P= 0.008). The P value for the ≤ 1w vs. 1-2w group was 0.327, for the 1-2w vs. >2w group was 0.149, for the ≤ 1w vs. >2w group was 0.002. (b) The estimated 5-year OS rates in the Ruijin & SJTU cohort for the ≤ 1w, 1-2w, and > 2w groups were 96.2%, 88.1%, and 91.3%, respectively (P< 0.001). The P value for the ≤ 1w vs. 1-2w group was < 0.001, for the 1-2w vs. >2w group was 0.560, for the ≤ 1w vs. >2w group was 0.004.
